# Knockout of NMDA-receptors from parvalbumin interneurons sensitizes to schizophrenia-related deficits induced by MK-801

**DOI:** 10.1038/tp.2016.44

**Published:** 2016-04-12

**Authors:** A M Bygrave, S Masiulis, E Nicholson, M Berkemann, C Barkus, R Sprengel, P J Harrison, D M Kullmann, D M Bannerman, D Kätzel

**Affiliations:** 1 Department of Experimental Psychology, University of Oxford, Oxford, UK; 2 Institute of Neurology, University College London, London, UK; 3 Department of Psychology, University of Landau, Landau, Germany; 4 Department of Pharmacology, University of Oxford, Oxford, UK; 5 Max-Planck Institute for Medical Research, Heidelberg, Germany; 6 Department of Psychiatry, University of Oxford, and Oxford Health NHS Foundation Trust, Warneford Hospital, Oxford, UK; 7 Institute of Applied Physiology, Ulm University, Ulm, Germany

## Abstract

It has been suggested that a functional deficit in NMDA-receptors (NMDARs) on parvalbumin (PV)-positive interneurons (PV-NMDARs) is central to the pathophysiology of schizophrenia. Supportive evidence come from examination of genetically modified mice where the obligatory NMDAR-subunit GluN1 (also known as NR1) has been deleted from PV interneurons by *Cre*-mediated knockout of the corresponding gene *Grin1* (*Grin1*^*ΔPV*^ mice). Notably, such PV-specific GluN1 ablation has been reported to blunt the induction of hyperlocomotion (a surrogate for psychosis) by pharmacological NMDAR blockade with the non-competitive antagonist MK-801. This suggests PV-NMDARs as the site of the psychosis-inducing action of MK-801. In contrast to this hypothesis, we show here that *Grin1*^*ΔPV*^ mice are not protected against the effects of MK-801, but are in fact sensitized to many of them. Compared with control animals, *Grin1*^*ΔPV*^mice injected with MK-801 show increased stereotypy and pronounced catalepsy, which confound the locomotor readout. Furthermore, in *Grin1*^*ΔPV*^mice, MK-801 induced medial-prefrontal delta (4 Hz) oscillations, and impaired performance on tests of motor coordination, working memory and sucrose preference, even at lower doses than in wild-type controls. We also found that untreated *Grin1*^*ΔPV*^mice are largely normal across a wide range of cognitive functions, including attention, cognitive flexibility and various forms of short-term memory. Taken together these results argue against PV-specific NMDAR hypofunction as a key starting point of schizophrenia pathophysiology, but support a model where NMDAR hypofunction in multiple cell types contribute to the disease.

## Introduction

Schizophrenia is a common and debilitating disease with a wide spectrum of symptoms. While positive symptoms such as hallucinations and delusions are central to its diagnosis, a large share of the disease burden results from negative and cognitive symptoms, such as social withdrawal, decreased motivation, and anhedonia, or deficits in working memory, attention and cognitive flexibility, respectively.^1^ Positive symptoms are related to excessive dopaminergic signaling and can be moderated accordingly by dopamine receptor antagonists. Negative and cognitive symptoms, however, do not reliably respond to neuroleptic treatment, and correlate with poor outcome.^2^

The observation that acute pharmacological blockade of NMDA-type glutamate receptors (NMDARs) induces positive, as well as cognitive and negative symptoms of schizophrenia in healthy individuals^3, 4^ has put NMDAR hypofunction at the center of much current thinking on schizophrenia pathogenesis, primary to dopaminergic hyperfunction.^5, 6^ The counter-intuitive finding that blockade of excitatory NMDARs, by a low dose of antagonist, induces an increase in local neural activity has led to the hypothesis that it is NMDARs on inhibitory interneurons that are preferentially affected by NMDAR blockers, causing a net disinhibition of principal cell activity.^5, 7, 8, 9^ Furthermore, post-mortem findings have implicated interneurons positive for parvalbumin (PV) in the disease process.^10, 11^ Consequently, the hypothesis that NMDAR hypofunction on PV interneurons is at the core of the pathophysiology of schizophrenia has been advanced in multiple versions.^6, 12, 13, 14^ A dominant model is that the resulting net disinhibition of principal cell activity increases the excitatory output of the ventral hippocampus, which could then lead to a dopaminergic hyperfunction, via polysynaptic loops through the basal ganglia.^6^

To test this PV-interneuron-NMDAR hypofunction hypothesis, several laboratories have generated genetically modified mice with conditional knockout of the obligatory GluN1 subunit of the NMDA-receptor in PV interneurons, although with varying degrees of specificity (*Grin1*^*ΔPV*^).^15, 16, 17, 18^ The behavioral phenotypes of these mice, on tests that might be considered relevant to schizophrenia, however, are highly variable across studies, and in many cases indistinguishable from controls.^17, 18, 19, 20^ Nevertheless, two studies have reported that NMDAR deletion in PV interneurons prevents the hyperlocomotion-inducing actions of the non-competitive NMDAR antagonist, MK-801.^15, 17^ The induction of hyperlocomotion by MK-801 (as well as by pro-dopaminergic drugs such as amphetamine) represents the most widely used animal model of positive symptoms of schizophrenia. This gains face validity from the observation that these drugs induce psychosis in healthy humans, and that both positive symptoms in patients and drug-induced hyperlocomotion in mouse models are attenuated by neuroleptic drugs. The absence of MK-801-induced hyperlocomotion in *Grin1*^*ΔPV*^ mice supports the hypothesis that NMDAR antagonists produce their psychotic effect specifically by blocking NMDARs on PV interneurons, and by extension constitutes some of the strongest evidence in favor of the PV-interneuron-NMDAR hypofunction hypothesis of schizophrenia.

However, this conclusion requires further scrutiny for several reasons: first, if it is specifically the blockade of NMDARs on PV interneurons that induces hyperlocomotion and other schizophrenia-relevant phenotypes, then why does knockout of NMDARs in such neurons not cause reliable cognitive deficits and hyperlocomotion in the first place?^15, 17, 18, 20^ Second, locomotor activity is a rather non-specific assay of behavior and its automated analysis via infrared beam-break counts may be affected by sedation and various other behaviors such as stereotypies. Third, studies of genetically modified mice in which NMDARs have been selectively ablated from principal cells in the forebrain have revealed robust hyperactivity, as well as deficits in tests of cognition that might be relevant to schizophrenia.^21, 22, 23, 24, 25, 26, 27, 28, 29^ Taken together, these observations call for closer scrutiny of the evidence that NMDARs solely on interneurons mediate the behavioral effects of NMDAR blockade.

We therefore undertook a detailed re-analysis of schizophrenia-related behavioral phenotypes in *Grin1*^*ΔPV*^ mice. We also re-examined the effects of MK-801 on locomotor activity in these animals, as well as on other phenotypes associated with the drug. We show that untreated *Grin1*^*ΔPV*^ mice are largely normal across a wide range of schizophrenia-related behavioral tests. Furthermore, we show that rather than being protected against the effects of MK-801 *Grin1*^*ΔPV*^ mice are in fact sensitized to its actions.

## Materials and methods

### Subjects

To achieve NMDAR ablation specifically in PV interneurons, we crossed a widely used and validated PV-*Cre*-driver line^30^ with the *Grin1*-2lox line used in the study that showed, as far as can be compared, the strongest behavioral deficits overall.^16^ This *Grin1*-2lox line features a smaller distance (3.3 kB) between the loxP sites compared with other lines used for conditional *Grin1* knockout,^25^ and is therefore expected to lead to NMDAR ablation earlier in development and hence stronger phenotypes.^15^ Mice harboring one copy of *Cre* and two copies of the *Grin1*-2lox allele were used as PV-interneuron-specific knockouts (hereafter *Grin1*^*ΔPV*^ or knockouts). Littermates with no copy of *Cre*, but one (*Grin1*^loxP/+^) or two (*Grin1*^loxP/loxP^) copies of the *Grin1*-2lox allele (hereafter *Grin1*-2lox or controls) were used as the control group. We confirmed, that the PV-*Cre* driver line we used achieves near-complete coverage of the neocortical PV-cell population (96% on average) and very comprehensive targeting of the hippocampal PV-neurons (84%) by 2 months of age, producing a significantly reduced NMDAR-current in PV-cells of knockouts (see Supplementary Figure S1), in agreement with previous data.^17^ Hence, mice were tested from the age of 2 months onwards to ensure NMDAR hypofunction across the PV-cell population.

### Behavioral assessment of *Grin1*^*ΔPV*^ mice

To investigate the causal involvement of NMDAR hypofunction, specifically in PV-cells, in schizophrenia-related behavioral phenotypes, we used two complementary approaches: first, we performed an extensive behavioral assessment of *Grin1*^*ΔPV*^ mice, and second we tested the impairments induced by pharmacological NMDAR blockade with MK-801 in control and *Grin1*^*ΔPV*^ mice.

Male and female *Grin1*^*ΔPV*^ mice and their littermate controls were assessed on a battery of behavioral tests related to schizophrenia. This included novelty-induced hyperlocomotion, pre-pulse inhibition (PPI) and habituation of startle response, spatial working memory (rewarded alternation) in the T-maze, spontaneous spatial novelty preference in the Y-maze, novel-object recognition, social memory, reversal learning in the plus maze, the five-choice serial reaction time task (5-CSRTT) and sucrose preference. Tests of anxiety—the elevated plus maze, light–dark box and hyponeophagia—were also conducted. Detailed methods for each test are provided in Supplementary Materials and Methods.

### The effects of MK-801 in *Grin1*^*ΔPV*^ mice

In addition, we also assessed the effects of the NMDAR antagonist MK-801 (dizocilpine maleate) on behavioral performance and *in vivo* electrophysiology in *Grin1*^*ΔPV*^ mice and controls. Mice were injected i.p. with MK-801 (dose range 0.1–0.5 mg kg^−1^) or saline vehicle prior to assessment of locomotor activity in photocell activity cages. Behavioral scoring was also used to quantify the presence of stereotypy, catalepsy and ataxia during assessment of locomotor activity (see Results). We also investigated the effects of MK-801 or vehicle injection in *Grin1*^*ΔPV*^ mice and controls on the accelerating rotarod test of motor coordination, spatial working memory (rewarded alternation on the elevated T-maze), a sucrose preference test of anhedonia and during *in vivo* recording of local field potentials in mPFC (see Supplementary Materials and Methods for full details).

## Results

### Behavioral assessment of *Grin1*^*ΔPV*^ mice

**Figure 1:**
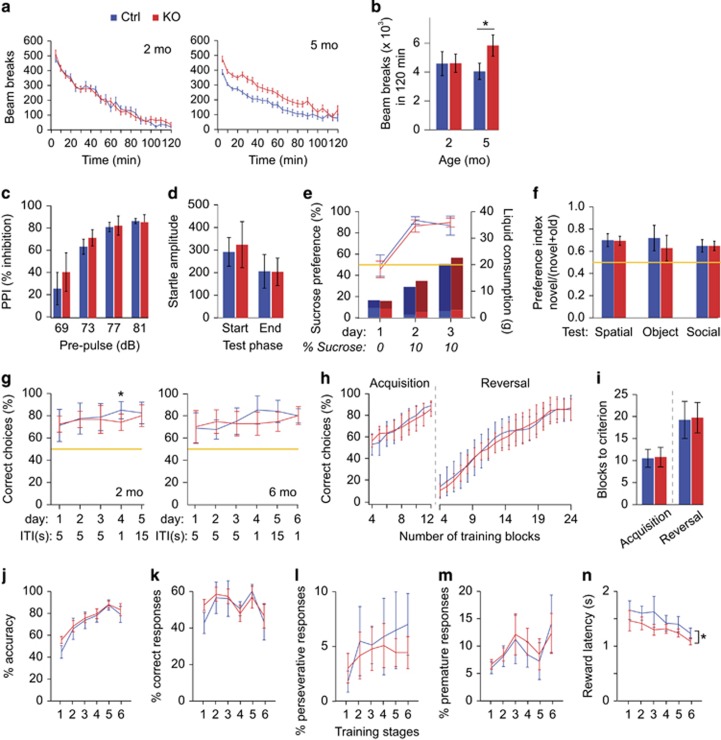
Schizophrenia-related endophenotypes and cognitive function in *Grin1*^*ΔPV*^ mice. (**a** and **b**) Novelty-induced hyperlocomotion: (**a**) Average beam-break counts over 2 h aggregated in 5-min bins for cohorts of 2 months (left) and 5 months (right) age (Error bars: s.e.m.). (**b**) Average total beam breaks for both age groups as indicated. Different age groups are distinct cohorts. (**c**) Average pre-pulse inhibition expressed as % for the individual dB-levels of pre-pulse. (**d**) Average absolute response to the startle-pulse (120 dB) at start and end of the test sequence, showing habituation to the startle-pulse over time. (**e**) Sucrose preference for 3 consecutive days during which two water bottles were presented in the first day, and one water and one bottle with 10% sucrose solution was offered on 2 consecutive days. Line graphs (left axis) represent the preference for the bottle that contained sucrose on days 2–3, while bar graphs show the absolute consumption of water (light blue/red) and sucrose (dark blue/red; right axis). (**f**) Short-term memory for spatial, non-spatial or social stimuli as indicated, assessed by spatial novelty preference (Y-maze), novel-object recognition and the three-chamber social memory task, respectively. Preference scores were calculated as the total time spent with the novel stimulus divided by the time spent with both stimuli combined. The yellow line indicates equal preference (chance levels). (**g**) Spatial working memory: correct choices (alternation of choice arm from sample to choice run) in the rewarded alternation paradigm in the T-maze are shown for training days 1–3 with 5-s intra-trial interval (ITI), and test days with 1 s and 15 s ITI. ITI-numbers are written in black to indicate round-robin and gray for massed trails. The same cohort was trained and tested twice at 2 months (left) and 6 months (right) of age. The yellow line indicates chance level. (**h** and **i**) Spatial reference memory and cognitive flexibility: (**h**) Percent of correct choices in the previous 20 trials in the plus maze plotted in intervals of 5 trials during acquisition and reversal learning. (**i**) Average number of blocks needed to reach criterion (85% correct in the previous 20 trials). (**j–n**) Averaged measures from the first 2 sessions in each of the 6 training stages mice were taken through in the 5-CSRTT (see Supplementary Methods for details): (**j**) % accuracy, (**k**) % correct responses, (**l**) % perseverative responses, (**m**) % premature responses and (**n**) reward latency. Blue, controls; red, knockouts. Asterisks indicate significant differences at *P<*0.05 measured by the appropriate statistical test (see Supplementary Table S1 for details). Error bars indicate 95% confidence intervals except were indicated otherwise. Ctrl, control; KO, knockout.

NMDAR hypofunction in hippocampal PV interneurons has been proposed to disinhibit hippocampal principal cell output and consequently induce over-activity of downstream dopaminergic neurons, causing both enhanced locomotor activity and reduced PPI.^6^ Previous studies have, however, failed to find such predicted phenotypes in *Grin1*^*ΔPV*^ mice.^15, 17, 20^ We re-examined locomotor activity in two cohorts, at different ages. We observed a significantly increased locomotion in knockouts at around 5 months (*P=*0.001) but not around 2 months of age (*P=*0.78; age–genotype interaction: *P=*0.02, analysis of variance (ANOVA); Figures 1a and b; see Supplementary Table S1 for statistical details on this and all subsequent tests). We next tested inhibition of startle responses at four pre-pulse intensity levels and found no evidence of a PPI deficit (*P=*0.46, effect of genotype, Figure 1c), nor abnormal habituation of the startle response across the session (*P=*0.527, genotype–phase interaction, repeated measures ANOVAs; Figure 1d, 5 months of age).

Schizophrenia may entail flattening of affect, including a decreased sense of pleasure (anhedonia) and an increased co-morbidity with anxiety disorders.^31^ Using the preference for a rewarding stimulus (10% sucrose solution) as a measure of hedonic motivation we found no effect of genotype (*P=*0.963, ANOVA; Figure 1e), in line with previous evidence.^32^ Unconditioned anxiety, in contrast to expectation, was mildly decreased in young knockouts when measured in the elevated plus maze but appeared largely normal in two other tests (Supplementary Figure S2).

Previous studies have reported impairments in *Grin1*^*ΔPV*^ knockout mice on cognitive tasks, such as working/short-term memory,^15, 16, 17^ although others have failed to detect such deficits.^18, 20^ We therefore assessed our *Grin1*^*ΔPV*^ mice in a battery of cognitive tests. We first assessed short-term memory for objects (novel-object recognition), and for spatial (spatial novelty preference, Y-maze) and social (three-chamber social memory paradigm) stimuli. *Grin1*^*ΔPV*^ mice showed a robust preference for the novel stimulus in all paradigms, indicating a memory for the familiar stimulus encountered during the sample trial (*P<*0.05, one-sample *t*-test against chance level of 0.5), and did not differ significantly from controls (*P>*0.2, ANOVA; Figure 1f, Supplementary Table S1).

Likewise, in the T-maze test of spatial working memory, knockouts performed well above chance levels in all blocks of testing (*P<*0.0005; one-sample *t*-test against 0.5; Figure 1g). Across the three different protocols tested (see Figure 1g), there was no significant main effect of genotype at either 2 (*P=*0.393) or 6 (*P=*0.232) months of age (repeated measures ANOVA). A previous study reported a working memory deficit when switching to a very short intra-trial interval of 1 s applied in massed trials.^17^ We therefore reproduced such a protocol-switch on the fourth day at both ages (2 and 6 months), and indeed found a significantly decreased performance in knockouts (*P=*0.004, repeated measures ANOVA across both ages). This effect of genotype, however, did not reach statistical significance at 6 months of age alone (*P=*0.084, *t*-test), which prompted us to repeat that 1-s protocol on day 6 of that training phase at 6 months. *Grin1*^*ΔPV*^ and control mice performed equally during that repetition (*P=*0.959, *t*-test; Figure 1g). Therefore, this subtle and transient decrease in spatial working memory performance is unlikely to reflect a robust impairment of working memory processes, but may instead reflect increased sensitivity to a change in testing protocols.

Impaired cognitive flexibility and attention, as well as increased perseveration are often considered hallmarks of schizophrenia. We used reversal learning in an appetitively motivated, discrete-trial, spatial choice task in an enclosed plus maze to measure cognitive flexibility. In line with a previous study which assessed spatial learning in the water maze,^17^ neither acquisition nor reversal of this associative memory were impaired (*P>*0.6, *t*-tests for number of trials to reach criterion; *P>*0.8 for main effects of genotype across blocks, repeated measures ANOVAs; Figures 1h and i). We also assessed performance on the 5-CSRTT, which is considered analogous to the continuous performance test that is often used to assess attentional performance in patients with schizophrenia.^33^ We trained mice through six stages during which the duration of the visual stimulus was gradually reduced to 1 s. There was no effect of genotype on measures of attention (% accuracy, % correct), perseveration (% repeated correct responses) or impulsivity (% premature responses; Figures 1j–m; see Supplementary Table S1 for statistics).^33^ *Grin1*^*ΔPV*^ mice were, however, faster to collect rewards during the task, which could reflect increased locomotor activity or increased motivation (Figure 1n).

In summary, despite the fact that our test battery covers a large—though not complete—range of schizophrenia-related deficits, *Grin1*^*ΔPV*^ mice displayed a surprising lack of behavioral aberrations, except for a mild hyperactivity at 5 months of age. It is imaginable that such a lack might—at least in part—be due to some compensation of NMDAR hypofunction by increasing expression of other excitatory channels over time. Therefore, we next conducted a complementary analysis of the involvement of PV-NMDARs in schizophrenia-related deficits assessing the effects of the non-competitive NMDAR antagonist, MK-801, in knockout mice and controls: If the lack of phenotype is due to compensation, while the hypothesis that the induction of schizophrenia-related deficits by MK-801 is largely mediated by action of the drug on PV-NMDARs holds (see Introduction), our knockouts should be protected against its effect.

### *Grin1*^*ΔPV*^ mice are not protected from the effects of MK-801 on locomotor activity

Two pioneering studies on interneuron-specific NMDAR-knockout mice reported that MK-801 fails to induce hyperlocomotion normally observed in wild-type mice.^15, 17^ We re-examined this issue (see Introduction) by combining locomotor activity monitoring with detailed, on-line behavioral scoring and tested three doses of MK-801 (0.1, 0.2 and 0.5 mg kg^−1^).

**Figure 2:**
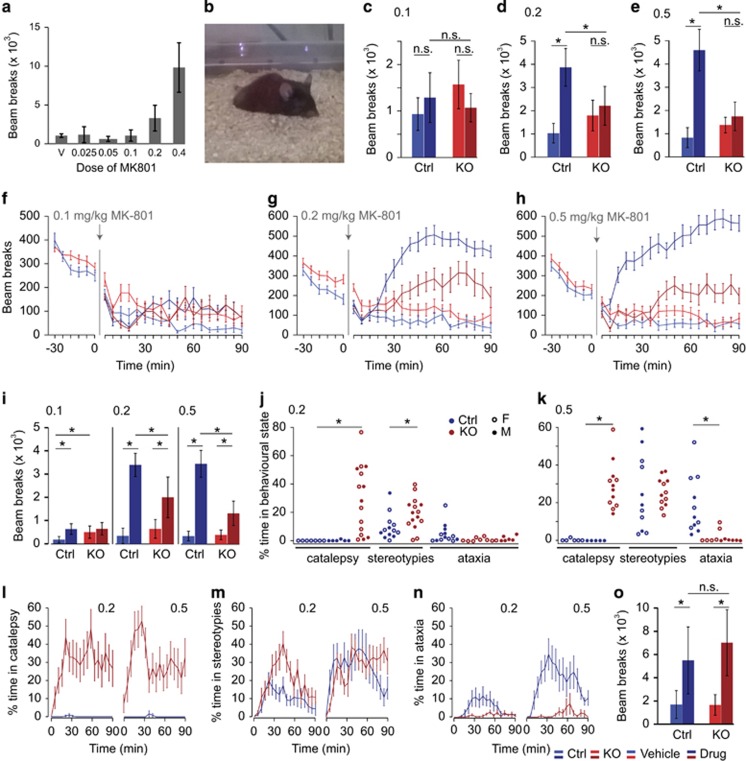
Effect of MK-801 on locomotor activity. (**a**) Dose–response curve of total beam breaks in 90 min post injection for rising doses of MK-801 (0.025 to 0.4 mg kg^−1^, *n*=4 per dose) in male C57bl6 mice. (**b**) Knockout mouse in MK-801-induced catalepsy (see also Supplementary Videos 2, 3, 4). (**c–e**) Total beam breaks for 60 min post injection for 0.1 (**c**), 0.2 (**d**) and 0.5 (**e**) mg kg^−1^ MK-801 vs vehicle. Asterisks indicate significant differences between MK-801-injected groups and relevant control groups (*P<*0.05, ANOVA). Note that beam-break counts in vehicle-injected KO mice are significantly higher compared with vehicle-injected controls in all three panels (indication omitted for clarity). (**f–h**) Average number of beam breaks over 30 min before and 90 min after injection of vehicle/MK-801 in 5-min intervals. (**i**) Total beam breaks for 60–90 min post injection of 0.1 (left), 0.2 (middle) and 0.5 (right) mg kg^−1^ MK-801 vs vehicle. Annotation as in **c–e**. (**j** and **k**) Counts for the three principle categories of behavior induced by 0.2 (**j**) and 0.5 (**k**) mg kg^−1^ MK-801: catalepsy, stereotypies and ataxia as indicated (see Results for details of scoring) for each animal expressed as % of total experimental time post injection (90 min). Solid circles, males; open circles, females. Asterisks indicate significant pair-wise differences (*P<*0.05, MW *U*-test or *t*-test as appropriate). (**l–n**) % time spent in states of catalepsy (**l**), stereotypies (**m**) and ataxia (**n**) plotted in 5-min intervals for 90 min after injection of 0.2 (left) or 0.5 (right) mg kg^−1^ MK-801. (**o**) Total beam-break counts over 90 min after injection of 0.2 mg kg^−1^ amphetamine (dark blue, dark red) or vehicle (light blue, light red). Asterisks indicate significant differences between drug-injected groups and relevant control groups (*P<*0.05, ANOVA). Error bars represent s.e.m. in line graphs (**f**–**h**) and otherwise 95% confidence intervals. All experiments were conducted as between-subjects designs. MK-801 (dark blue, dark red) or vehicle (light blue, light red) indicated in control (blue) and knockout (red) mice. Gray numbers represent doses of MK-801 for each panel. ANOVA, analysis of variance; Ctrl, control; KO, knockout; NS, not significant.

In wild-type mice, MK-801 produced a dose-dependent increase in spontaneous locomotion as expected (Figure 2a). We classified other MK-801-induced motor aberrations into three types: (1) stereotypies, mainly consisting of circling but also repetitive head-shaking, face-washing, grooming or jumping; (2) ataxia or 'tumbling' characterized by repeated loss of balance while walking or rearing (Supplementary Video 1); and (3) catalepsy, corresponding to extended periods lasting up to several minutes of complete immobility. From ~15 min post injection onwards, such episodes of immobility were distinguishable from periods of normal rest or sleep in vehicle-injected animals because mice lacked the typical rounded posture standing on four legs seen in control mice, and instead exhibited a more stretched posture with the head and/or body lying directly on the floor, and with legs stretched out (Figure 2b; Supplementary Videos 2-4).

We first confirmed previous reports^15, 17^ that in *Grin1*^*ΔPV*^ mice MK-801 almost completely failed to increase locomotion when measuring aggregate beam-break counts over 60 min post injection (drug–genotype interactions for MK-801 doses in mg kg^−1^: 0.1: *P=*0.023, 0.2: *P=*0.001, 0.5: *P<*0.0005; effects of drug: 0.1: *P=*0.801, 0.2 and 0.5: *P<*0.0005, ANOVA; Figures 2c–e; see Supplementary Table S2 for details on this and all subsequent statistics). At none of the doses tested did MK-801 significantly increase the total beam-break counts over the first 60 min in knockouts relative to vehicle (*P>*0.2 for 0.2 and 0.5 mg kg^−1^; *P=*0.074 for 0.1 mg kg^−1^ with MK-injected knockouts displaying lower beam breaks, ANOVA; Figures 2c–e). In contrast, in controls total locomotor activity was profoundly increased by MK-801 at the two higher doses (*P<*0.0005, ANOVA) relative to vehicle.

However, closer inspection of the animals' behavior demands a re-interpretation of this observation. First, when measuring aggregate beam-break counts over the last 30 min only (60–90 min post injection), MK-801 actually induced significant hyperlocomotion in knockouts (*P<*0.005 for effects of 0.2 and 0.5 mg kg^−1^ MK-801 within knockout group, ANOVA), albeit less than in control mice (*P<*0.02 for effects of genotype within MK-801-injected mice; *P<*0.01 for drug–genotype interaction, ANOVAs; Figure 2i, see also Figures 2f–h and ref. 15).

Second, manual scoring revealed that the lower beam-break counts in MK-801-injected *Grin1*^*ΔPV*^ mice were mainly accounted for by extended periods of catalepsy (Figures 2j–l). At higher doses (0.2 and 0.5 mg kg^−1^) catalepsy was observed in every knockout mouse, but was virtually absent in wild-type mice (*P<*0.0005, Mann–Whitney (MW) *U*-test; Figures 2j–l). During blind-to-genotype observations, only 2 out of 24 wild-type mice exhibited a single episode each classed as cataleptic. Indeed, the genotype could be predicted virtually without error from this recurring MK-801-induced trait.

Third, knockout mice spent more time engaged in stereotypies at 0.2 mg kg^−1^ compared with MK-801-injected controls (*P=*0.016, MW *U*-test, Figures 2j and m). Interestingly, however, knockouts displayed very little MK-801-related disturbance of balance (ataxia) in the locomotor activity cages, while all controls were affected (0.2 mg kg^−1^: *P=*0.016, 0.5 mg kg^−1^: *P<*0.0005; MW *U*-tests, Figures 2j, k and n).

We next tested whether the reduced locomotor activity in *Grin1*^*ΔPV*^ mice following MK-801 could indicate a general lack of sensitivity to psychostimulants by assessing the locomotor response to amphetamine, a drug that directly increases dopaminergic signaling. However, knockouts were at least as responsive to its locomotor activity-inducing effect as controls (effect of drug: *P<*0.0005, drug–genotype interaction: *P=*0.41, ANOVA; Figure 2o).

### *Grin1*^*ΔPV*^ mice are sensitized to MK-801-induced impairments of coordination and cognition

These results suggest that NMDAR blockade may still induce some hyperlocomotion and stereotypy, that is, potential correlates of psychosis and habitual behavior, in *Grin1*^*ΔPV*^ knockout mice, and that following MK-801 injection beam-break counts in knockouts are only lower than in controls because of drug-induced catalepsy in the former. To test this interpretation further, we assessed motor performance on the accelerating rotarod in both genotypes at an intermediate dose of the drug (0.15 mg kg^−1^ in females, 0.2 mg kg^−1^ in males). If knockouts are really protected from motor effects of NMDAR blockade as suggested,^15, 17^ then they should perform at least as well on the rotarod as MK-801-injected controls.

**Figure 3:**
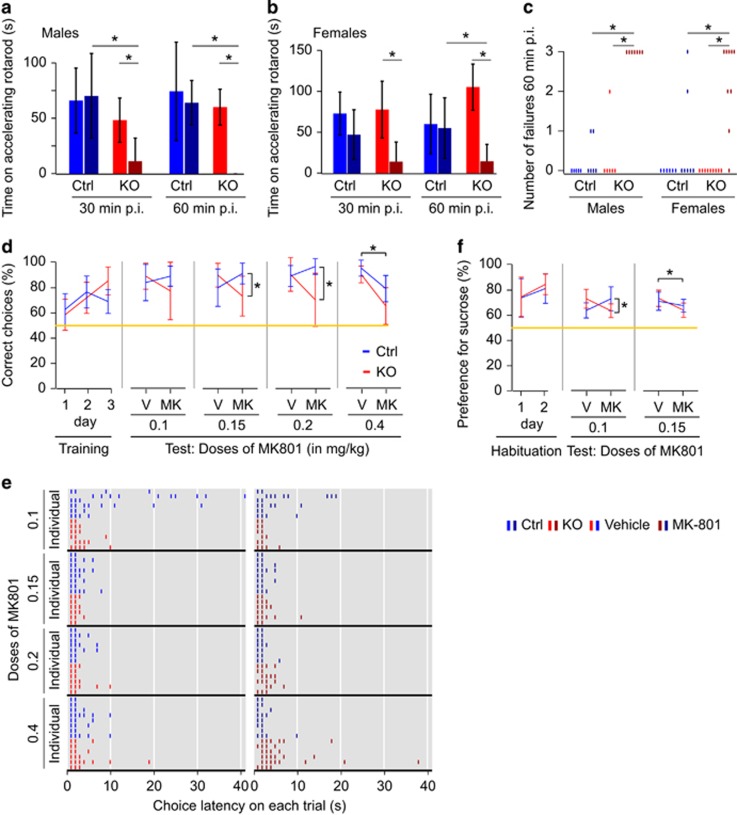
MK-801-induced impairment of coordination, working memory and sucrose preference. (**a–c**) MK-801-induced impairment of motor coordination on the rotarod. (**a** and **b**) Time the males (**a**) or females (**b**) managed to remain on the accelerating rotarod at 30 min (left) and 60 min (right) after the injection of MK-801 (dark blue, dark red; 0.15 mg kg^−1^ in females; 0.2 mg kg^−1^ in males) or vehicle (light blue, light red). Asterisks indicate significant differences at *P<*0.05 (ANOVA). (**c**) The total number of failures (falling of the rod before acceleration) on three attempts at 60 min after injection (color code as in a). Asterisks indicate significant differences at *P<*0.05 (MW *U*-test). (**d**) Correct choices (% out of 10 trials per block) on the T-maze rewarded alternation task are shown for the first three blocks of training, as well as for MK-801- and matched vehicle-trials at increasing dose of MK-801 as indicated. The asterisks indicate a significant drug–genotype interaction at 0.15 and 0.2 mg kg^−1^ (only knockouts are affected), or a significant effect of drug at 0.4 mg kg^−1^ (both genotypes are affected); repeated measures ANOVA followed by *t*-tests. (**e**) Latencies on the choice trials (measured from insertion into the start arm until arrival in the choice arm to an accuracy of 1 s, *x*-axis) vehicle (left) and corresponding MK-801 (right) trials are plotted for every trial of each animal and dose (vertical axis). Each animal successfully ran 10 out of the 10 trials in each block, but data points from trials with identical latencies are plotted on top of one another. (**f**) Percent sucrose-solution (1%) consumption of total liquid consumption is plotted for 2 habituation days (left), as well as for vehicle (V)- and MK-801 (MK)-trials at the indicated doses (in mg kg^−1^). The asterisks indicate significant drug–genotype interaction at 0.1 mg kg^−1^ (only knockouts are affected), or a significant effect of drug at 0.15 mg kg^−1^ (both genotypes are affected); repeated measures ANOVA followed by *t*-tests. In all plots blue lines represent controls, red lines knockouts, with MK-801 (dark blue, dark red) or vehicle (light blue, light red) as indicated. Yellow lines indicate chance levels (50%). Error bars represent 95% confidence intervals. All experiments were conducted as within-subjects designs. ANOVA, analysis of variance; Ctrl, control; KO, knockout.

In both males and females, the time on the accelerating rotarod was significantly lower for MK-801-injected knockout mice compared with MK-801-injected controls, as well as compared with vehicle-injected knockouts (*P<*0.01, *t*-tests; drug–genotype interaction: *P<*0.01, ANOVA; 60 min post injection; Figures 3a–c). The number of failures, that is, occasions of falling off the rotarod before acceleration even started, was significantly higher in MK-801-injected knockouts than in both MK-801-injected controls and vehicle-injected knockouts (*P<*0.05, 60 min post injection, MW *U*-test; Figure 3c). The rotarod test thus provides further evidence that *Grin1*^*ΔPV*^ mice are more sensitive to MK-801-mediated motor disturbances than controls.

Acute administration of NMDAR antagonists not only induces locomotor effects but also impairments in cognition,^34, 35, 36, 37^ including in spatial working memory on the T-maze.^38^ We trained a cohort of *Grin1*^*ΔPV*^ mice and controls on the rewarded alternation T-maze paradigm, which we had used previously (Figure 1g). After training each animal to criterion, we assessed mice at doses of 0.1, 0.15, 0.2 and 0.4 mg kg^−1^ MK-801, starting at 30 min post injection. For each individual dose, we included a separate within-subjects vehicle control condition (fully counterbalanced). We found an MK-801-induced impairment for both genotypes only at a dose of 0.4 mg kg^−1^ (main effect of drug at 0.4 mg kg^−1^: *P<*0.0005; *P>*0.1 at other doses; drug–genotype interaction at 0.4 mg kg^−1^: *P=*0.336; repeated measures ANOVA, Figure 3d). At the lowest dose (0.1 mg kg^−1^) in turn there was no significant impairment (*P>*0.1 for effects of drug, genotype and drug–genotype interaction, repeated measures ANOVA). At intermediate doses (0.15 and 0.2 mg kg^−1^), however, *Grin1*^*ΔPV*^ knockouts were already significantly impaired compared with vehicle condition (*P<*0.05, paired samples *t*-tests) while controls were not (drug–genotype interaction: *P<*0.02, repeated measures ANOVA for both drug doses separately; Figure 3d). There was no overall effect of genotype during the training phase or on vehicle days, indicating equivalent baseline performance of knockouts and controls (*P>*0.5, repeated measures ANOVAs), consistent with our earlier observations (Figure 1g). Thus, *Grin1*^*ΔPV*^ mice were sensitized to the amnestic effects of MK-801. Importantly, we did not observe catalepsy or any other gross motoric impairment during spatial working memory testing with the drug in either genotype in food-deprived mice that would have resulted in a failure to travel swiftly through the maze to the food wells: choice latencies remained largely in the normal range (under 10 s), even at the highest dose of MK-801 in the knockouts (Figure 3e).

Finally, to assess the generality of this finding, we also examined the reduction in sucrose preference seen with MK-801, which might relate to the anhedonia-like aspect of negative symptoms seen in some patients with schizophrenia.^39^ We tested the preference for 1% sucrose solution after treatment with 0.1 and 0.15 mg kg^−1^ MK-801 during a 2.5 h post injection period. We found that at the lower dose of the drug (0.1 mg kg^−1^), only knockouts showed a significant decrease in sucrose preference (drug–genotype interaction: *P=*0.012; no effect of drug: *P=*0.965 or genotype: *P=*0.960, repeated measures ANOVA). In contrast, at the higher dose of MK-801, there was reduced sucrose preference in both genotypes (no drug–genotype interaction: *P=*0.276; significant main effect of drug: *P=*0.026, but not of genotype: *P=*0.798; repeated measures ANOVA; Figure 3f).

### MK-801 induces cortical delta-oscillations in *Grin1*^*ΔPV*^ mice

**Figure 4:**
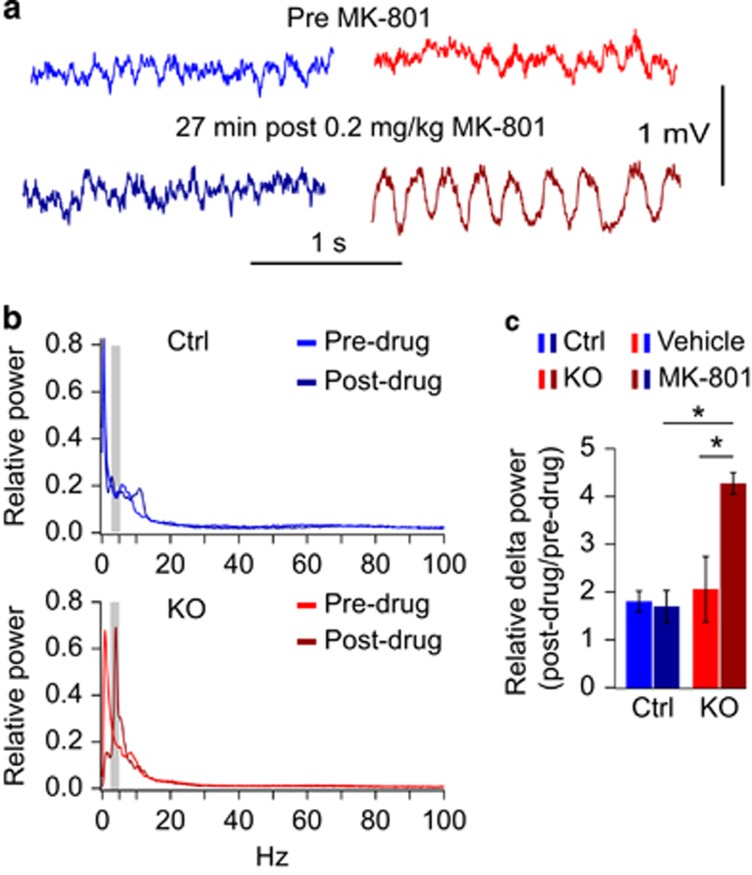
Induction of medial-prefrontal delta-oscillations by MK-801 in *Grin1*^*ΔPV*^ mice. (**a**) Example local field potential (LFP) traces of 2-s duration recorded in mPFC of control (left, blue) and knockout (right, red) mice before (top) and 27 min after (bottom) injection of 0.2 mg kg^−1^ MK-801. (**b**) Power spectra calculated for LFP recordings of 10 min, either immediately before (light blue/red) or 20–30 min after (dark blue/red) injection of MK-801 and normalized to total power spectral density. Gray bar highlights the area of 3–5 Hz containing the peak of the induced delta-oscillations and used for the analysis in **c**. (**c**) Power in the 3–5 Hz range normalized to total power spectral density and expressed as a ratio of 20–30 min to −10–0 min (baseline). Note that delta power generally increases over time in all conditions (ratio>1) including after vehicle injection (light blue/red, within-subject comparison), but only increases dramatically in MK-801-injected knockouts (dark red), not MK-801-injected controls. Error bars represent s.e.m., asterisk represents significant difference (see main text), *n=*3 per group.

The decrease of working memory already at intermediate doses of MK-801 in knockouts speaks to a perturbation of prefrontal or hippocampal circuits. To assess this further, we recorded local field potentials in medial-prefrontal cortex and CA1-hippocampus 10 min before and 20–30 min after injection of 0.2 mg kg^−1^ MK-801 or vehicle in freely moving mice. While local field potentials in control mice did not display apparent MK-801-induced changes in either of those areas, prefrontal cortex in *Grin1*^*ΔPV*^ mice became dominated by slow oscillations (Figure 4a). Power-spectral analysis confirmed the emergence of a peak at ~4 Hz (Figure 4b). The total relative power in the high delta range (3–5 Hz) increased significantly in *Grin1*^*ΔPV*^ mice treated with MK-801 compared with MK-801-injected controls (*P=*0.03 *t*-test), as well as compared with the vehicle condition (drug–genotype interaction: *P=*0.019, repeated measures ANOVA; Figure 4c). It is imaginable that engagement of prefrontal network activity by such dominant oscillations, which normally occur only during non-REM sleep,^40^ interferes with working memory processes.

## Discussion

### *Grin1*^*ΔPV*^ knockout mice do not display marked cognitive impairments

In the present study we have shown that, under baseline conditions, *Grin1*^*ΔPV*^ mice exhibit mild, age-dependent locomotor hyperactivity, but otherwise little, if any, aberrant phenotype across a wide battery of cognitive tasks that might be considered relevant to schizophrenia, including measures of working or short-term memory, attention, PPI and cognitive flexibility. This cannot be explained by ineffectiveness of knockout as we combined a PV-*Cre* driver with comparatively high coverage of the PV-cell population (see Supplementary Figure S1) with a *Grin1*-2lox responder line that features a short distance between *loxP* sites (see Supplementary Table S3), which should facilitate NMDAR knockout.^15, 16^ Our data therefore question a circuit model in which PV-specific NMDAR hypofunction is a causal starting point of schizophrenia pathology.^6^ Failure to induce a robust phenotype by deletion of NMDARs in PV interneurons largely agrees with earlier studies using the same PV-*Cre* driver line (see Supplementary Table S3), which repeatedly failed to detect profound deficits in working memory (using various T-maze tasks), cognitive flexibility (using reversal learning in the water maze) or PPI.^17, 18, 20^ These results, however, stand in contrast with a study that used a different driver line, which targets only a subset of the brain's PV interneurons, mainly located in the hippocampus,^41, 42^ and revealed an impairment of short-term memory.^16^

### *Grin1*^*ΔPV*^ knockout mice exhibit increased sensitivity to MK-801

Considering that the behavior of *Grin1*^*ΔPV*^ mice was either normal or showed subtle alterations under baseline conditions, their clear divergence from wild-type behavior under MK-801-treatment is striking. The experimental logic of such experiments is that knockouts should be protected against MK-801-induced effects assuming these effects are caused by blocking NMDARs on PV interneurons in control mice.

Contrary to previous conclusions,^15, 17^ however, we found that *Grin1*^*ΔPV*^ mice are not protected against most behavioral effects of MK-801 that we tested. Instead they were even sensitized to the drug's action in several paradigms: knockouts displayed increased stereotypies, as well as strong catalepsy compared with drug-treated control mice. Moreover, MK-801 induced deficits in motor coordination, spatial working memory and sucrose preference at lower doses in *Grin1*^*ΔPV*^ mice than in controls indicating their sensitization to the drug. Even mild hyperlocomotion, a mouse correlate of psychosis, was evident with MK-801 in *Grin1*^*ΔPV*^ mice after 1 h post injection when episodes of catalepsy became less pronounced. Thus, NMDARs on PV interneurons are not the (primary) site of drug action for these behavioral effects of non-competitive NMDAR antagonists, and, by inference, the virtual lack of a phenotype in baseline behavior cannot simply be explained by compensatory mechanisms.

### Implications for neural circuit models of schizophrenia

In summary, our data argue against the hypothesis that NMDAR hypofunction specifically on PV interneurons is central to the psychotic and cognitive symptoms of schizophrenia. Instead, PV-NMDAR hypofunction appears to predispose or sensitize the circuit to some behavioral consequences of reduced NMDAR-signaling in other neurons in the circuit. PV-NMDAR hypofunction may thus be one of many potential risk factors of the disorder. The finding that some deficits are provoked by long-term social isolation stress in one interneuron-specific NMDAR-knockout line,^15, 19^ as well as by ageing as described here for hyperlocomotion, supports this risk-factor-model. Also, our conclusion is arguably more consistent with the data in schizophrenia itself:^43^ although several pathological studies have reported a reduced expression of NMDAR subunits, including in interneurons^44, 45^ there is little evidence that interneurons, let alone the PV subclass, are disproportionately affected.^46^ Further in line with our results, several other studies have found that targeting NMDAR knockout exclusively to specific excitatory cells of the forebrain may cause profound schizophrenia-related deficits, including impairments of short-term or working memory, sociability and PPI, as well as increased hyperlocomotion.^25, 26, 29, 47^

Instead a more complex framework, which can accommodate NMDAR hypofunction at more than one node in the network and can explain the interaction between these nodes, is necessary. At this stage one can only speculate about the nature of such an interaction. One possibility is, that NMDARs expressed by multiple cell types in cortical circuits collectively help to fine-tune the balance between excitation and inhibition (E/I balance): From this perspective, an NMDAR blockade in *Grin1*^*ΔPV*^ mice would equate to a depression of cortical network activity because excitatory drive is removed predominantly from excitatory cells, not from PV interneurons. Such depression, if occurring in motor-related areas, might be the cause of the catalepsy we observed. In wild-type mice, in contrast, excitatory drive is removed from inhibitory PV-neurons as well, thereby preventing such depression. A related possibility is the induction of an imbalance between cortical and thalamic circuits: delta-oscillations, which normally occur only during slow-wave sleep, can also be observed during the awake state in the ventromedial prefrontal cortex in patients with schizophrenia.^48^ It has been suggested, that these oscillations result from a hypofunction of NR2C-containing NMDARs in the thalamus, potentially in PV interneurons enriched in this region.^49^ This specification of the hypothesis is disproved by the fact that we could induce strong prefrontal delta-oscillations with MK-801 in mice in which NMDARs on PV-cells are missing. However, our data leave open the possibility that NMDAR blockade on other thalamic cell types causes a cortico-thalamic imbalance. A further possible correlate of the sensitization to impairment of motor coordination is an alteration of cerebellar circuits, which also contain PV interneurons. Besides, the schizophrenia-related role of the minority of excitatory PV-positive cells in the brain^50^ remains to be elucidated. Our approach demonstrates the utility of cell type-specific NMDAR-knockout mice as experimental tools for mechanistic investigation of the glutamate hypothesis of schizophrenia, even if such mice cannot be considered models of this disease.
