# Supplementary material for: Knockout of NMDA-receptors from parvalbumin interneurons sensitizes to schizophrenia-related deficits induced by MK-801
Source: Transl Psychiatry. 2016 Apr 12;6(4):e778–. doi: 10.1038/tp.2016.44 (PMC4872402; doi:10.1038/tp.2016.44)
Supplement: Supplementary Information [file tp201644x1.doc]

# Supplementary Information

# Knockout of NMDA-receptors from parvalbumin interneurons sensitizes to schizophrenia-related deficits induced by MK-801

***Authors:***

Alexei M. Bygrave^1^ , Simonas Masiulis^1^, Elizabeth Nicholson^2^, Michelle Berkemann^1,3^, Chris Barkus^4^, Rolf Sprengel^5^, Paul J. Harrison^6^, Dimitri M. Kullmann^2^*, David M. Bannerman^1^*, Dennis Kätzel^1,2,7^*

**^1^** Dept. of Experimental Psychology, University of Oxford, Oxford, UK

^2^ Institute of Neurology, University College London, London, United Kingdom

^3^ Dept. of Psychology, University of Landau, Germany

^4^ Dept. of Pharmacology, University of Oxford, Oxford, UK

^5^ Max-Planck Institute for Medical Research, Heidelberg, Germany

^6^ Dept. of Psychiatry, University of Oxford, and Oxford Health NHS Foundation Trust, Warneford Hospital, Oxford, UK

^7^ Institute of Applied Physiology, Ulm University, Ulm, Germany

*** Correspondence:**

Dimitri M. Kullmann, Institute of Neurology, University College London, Queen Square, WC1N 3BG, London, United Kingdom, d.kullmann@ucl.ac.uk

David M. Bannerman, Dept. Of Experimental Psychology, University of Oxford, 9 South Parks Road, OX1 3UD, Oxford, United Kingdom, david.bannerman@psy.ox.ac.uk

Dennis Kätzel, Institute of Applied Physiology, Ulm University, 89081 Ulm, Germany; dennis.kaetzel@uni-ulm.de

## Supplementary figures

**Supplementary Figure 1. Immunohistochemical analysis of the PV-*Cre* driver line used.**

(**a**) Example images from a neocortical section stained against Parvalbumin (PV, green) and showing intrinsic fluorescence from expressed tdTomato (red); merged image on the right. Projection through a 50 µm stack acquired with a confocal microscope from a section from a 10-week-old mouse. Scale bar, 20 µm. tdTomato is expressed after *Cre*-mediated recombination of a stop-floxed reporter gene from the ROSA26-locus (Ai9 line, B6.Cg-*Gt(ROSA)26Sor^tm9(CAG-tdTomato)Hze^*/J, Jackson Labs stock number 007909; Allen Institute, USA) in cells targeted by the PV-*Cre* line also used for selective ablation of GluN1 in subsequent experiments (B6;129P2-*Pvalb^tm1(cre)Arbr^*/J, Jackson Labs stock number 008069 ^1^).

(**b**) The number of tdTomato-positive cells was counted among the cells labelled by the PV-antibody in neocortex (NC), dorsal hippocampus (dHC) and ventral hippocampus (vHC), and is expressed as a percentage to indicate in how many PV-cells *Cre* has successfully led to excision of the floxed Stop sequence (recombination). In Neocortex, virtually the whole population of PV-cells is targeted by the PV-*Cre* driver line (95.6 ± 3.9 %; mean ± S.D.), while in hippocampus the coverage is somewhat lower (84.2 ± 8.3). A total of 11 stacks and 824 cells have been counted for neocortex, 6 stacks and 95 cells for dHC, and 8 stacks and 154 cells for vHC. Sections were prepared from two mice, aged 10 weeks. Displayed are mean values ± S.E.M.

(**c**) Same histological data as in (b) but analysed in reverse: The number of PV-positive cells was counted among the cells expressing tdTomato in neocortex (NC), dorsal hippocampus (dHC) and ventral hippocampus (vHC), and is expressed as a percentage to indicate how many cells in which *Cre* is/was active, are actually PV-cells (specificity). In neocortex and vHC the specificity is very high (95 %, mean), in dHC slightly lower (91 %).

(**d-f**) We created a triple transgenic mouse by crossing the PV-*Cre*::Ai9 line (see (a)) to our *Grin1*-2lox line, in order to create PV-NMDAR knockout mice, in which the PV-cells are labelled by tdTomato. Neocortical brain slices were obtained from these (KO) as well as PV-*Cre*::Ai9 control (Ctrl) animals (n = 2 mice each, 2 mo old), and PV-interneurons (n: 11 Ctrl, 8 KO) recorded in patch-clamp whole-cell configuration in voltage-clamp mode (see Supplementary Methods). (**d**) Example of a cell filled with biocytin during recording and stained with Streptavidin-Alexa488 subsequently (green) co-expressing tdTomato (red). (**e**) Example traces for the AMPA-receptor currents recorded at holding potentials of -70 mV (large downward slopes) and the NMDA-currents recorded at +40 mV holding potential (upward slope in Ctrl, flat line in KO). (**f**) Ratios of the amplitudes of NMDA and AMPA-currents are significantly smaller in KO compared to Ctrl cells (p = 0.035, Mann-Whitney-U-test, non-parametrically distributed). While 5/8 KO cells (63%) display ratios of < 0.1, only 2/11 Ctrl cells (18%) do. Yet, the remaining 3/8 KO cells still show moderate levels of NMDA-current, indicating that by that age (2 mo) the functional knockout is not complete yet, and is more akin to an NMDAR-hypofunction rather than an NMDAR-deletion.

(**g**) Novelty-induced hyperlocomotion – analysis of beam break counts in photocell cages for monthly age groups. *Rationale:* The KO of NMDARs is expected to reach completion around the transition from 2-3 mo (see also ref. (5)). The young cohort in our test of novelty-induced hyperlocomotion (Figure 1a, b) displayed the youngest age at testing within our data-set. The absence of the hyperlocomotion-phenotype which we uncover only at older age (5 mo and older) in that young cohort might be due to incomplete knockout of PV-NMDARs at this young age. In order to test for such a confound, we split the younger cohort analysing the mice of 3 mo of age separately from the mice of 2 mo of age. However, neither of these two groups displays any trend of hyperlocomotion in the knockouts, argueing against the hypothesis that an incomplete knockout underlies the age-dependent effect. Error bars, 95% confidence intervals.

**Supplementary Figure 2. Anxiety assessment in *Grin1^ΔPV^* mice.**

(**a**) Unconditioned anxiety in the elevated plus-maze: preference scores for the time spent on the open arm in distinct cohorts of 2 and 4 months of age as indicated. We found a significantly increased fraction of time spent in the open arm in 2 month-old KO mice (p = 0.025, asterisk), and a qualitatively similar, albeit non-significant, trend in 4 month-old animals (p = 0.220, for both cohorts combined p = 0.020, ANOVAs).The effect was not due to increased locomotion as determined by closed arm entries during the test (see Supplementary Table S1). No significant age-genotype interaction was found in this case (p = 0.58), as ageing increased anxiety in both genotypes (p = 0.008, ANOVA). The distinct age-groups are distinct cohorts.

(**b**) Hyponeophagia assessed by the latency to eat in an elevated, anxiogenic environment at 2 mo of age. While all knockouts (red) succeeded to eat within the given three consecutive trials of 120 s, 5 control mice (blue) did not and therefore received a maximum latency of 360 s. Although this could indicate a decreased anxiety in knockouts, the remainder of the controls showed a slightly decreased average latency-to-eat compared to knockouts, which complicates the interpretation. Using non-parametric testing (M.-W.-U-Test), no difference between the genotypes is indicated (p = 0.740).

(**c**) Preference for the light over the dark compartment in a Light-Dark-Box at 3 mo of age. No effect of genotype was found (p = 0.124, ANOVA).

Blue, controls; red, knockouts. Error bars represent 95 % confidence intervals. All tests were done in mixed-sex cohorts.

## Supplementary tables

**Supplementary Table 1**

##

**Analysis of baseline behaviour of *Grin1^ΔPV^* knockouts compared to controls.** Analysis was conducted for single protocol tests or combined multi-protocol tests (top), multiple stages within one test (middle) and age-dependent effects (bottom), where appropriate. Age is given in months. Abbreviations: PPI, pre-pulse inhibition; RM, repeated-measures ANOVA; Univ, Univariate ANOVA; MWU, Mann-Witney-U Test; Y, young cohort of the two; O, old cohort of the two; m, males; f, females; df, degrees of freedom in ANOVA; n, number of animals in the cohort.

**Supplementary Table 2**

**Analysis of MK-801-induced behaviour in *Grin1^ΔPV^* knockouts compared to controls.** Analysis was conducted across all sub-groups of each experiment (top) as well as in a pair-wise manner within genotypes and drug-groups (bottom). *Abbreviations:* LMA, locomotor activity; LMA(Amph), amphetamine-induced locomotor activity; RM, repeated-measures ANOVA; Univ, Univariate ANOVA; MWU, Mann-Witney-U Test; T, T-Test; m, males; f, females; df, degrees of freedom in ANOVA; n, number of animals in the cohort; numbers in brackets give dose of MK-801 used.

**Supplementary Table 3**

**Comparison of genetics of PV-interneuron-specific knockout lines in previous studies and the present one.** The driver and responder lines are indicated as well as the distance between lox-sites, which has been suggested to correlate with the speed of the stochastic process of the knockout of GluN1-subunits, and hence correlate negatively with deficit severity ^2^. All mouse lines were crossed into and tested in a C57bl/6 background, as far as can be inferred from the literature. References for the individual lines are as follows : Ppp1r2 ^2^, PV (Monyer) ^3,4^, PV (Arber)^1,5,6^ , Li ^7^, Tonegawa ^8^, Seeburg ^9^.

## Supplementary methods

**Animals**

Mice with a genomic insertion of *Cre*-recombinase into the 3’-UTR of the Parvalbumin-gene ^1^, (B6;129P2-*Pvalb^tm1(cre)Arbr^*/J, Jackson Labs stock number 008069), were crossed to Grin1-2lox mice in which the endogenous constitutive NMDAR-subunit *Grin1* is replaced by a homologous gene with loxP-sites flanking exons 11-18 ^9^, (B6.129-Grin1^tm1Rsp^/Kctt, EMMA stock number EM:09220). For genotyping, PCR-mediated amplification from genomic DNA-samples using primers 5‘ – TGT GTC CCT GTC CAT ACT CAA – 3‘ and 5‘ – AAC ACT GTG GAC CAG GAC TTG – 3‘ resulted in a 325 bp product for the Grin1-wildtype allele and in a 375 bp product for the floxed allele. *Cre*-recombinase was detected with generic primers 5’- CAC CCT GTT ACG TAT AGC CG – 3’ and 5- GAG TCA TCC TTA GCG CCG TA – 3’ resulting in a 330 bp product. Mice with one *Cre* and two floxed-*Grin1* alleles were deployed as *Grin1^ΔPV^* knockouts, while *Cre*-negative littermates with usually two and rarely one floxed-*Grin1* allele(s) were used as control mice. We aimed for a group size of 10 subjects for most experiments as recommended for behavioural testing ^10^, although some tests were done in much larger cohorts or replicated in multiple cohorts, respectively. Four male and three female cohorts of at least 20 mice each and at least 8 mice per genotype were bred for the described experiments. Mice were used between ages 2 and 13 mo, with MK-801-experiments conducted at the end of each sequence of experiments and locomotor and anxiety tests done early on in the test battery. Cognitive tasks were conducted before 7 mo of age. Animals were raised in IVC-cages and transferred to open-top cages at 1-2 mo of age, well before behavioural testing started. Both cage-types were filled with saw dust and equally enriched with card board houses, tubes and sizzle nest. Mice were group-housed at all times, except when isolation was required for experiments (sucrose preference testing). Behavioural testing was conducted during the light phase, except for assessments of sucrose preference. Experiments that involved manual scoring were conducted blind to genotype. All experiments conformed to the Animal (Scientific Procedures) Act 1986, UK, and the Local Ethical Review Committee at the University of Oxford.

**Immunohistochemistry**

PV-Ai9 mice were perfused with PBS followed by 4 % paraformaldehyd/PBS (PFA-PBS) and brains removed in PFA-PBS for 24h. 60 µm sections were cut on a vibratome in PBS, and sections washed in PBS 3-4 times. Sections were then incubated in 0.15 % Triton/TBS (TBS-T) for 20 min, 20 % horse serum/TBS-T for 1 hour, briefly washed in TBS-T, and then incubated for 48 h at 4
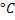
 with primary antibody (anti-Parvalbumin, raised in mouse, Swant, Switzerland) diluted 1:2000 in 2.5% horse serum/TBS-T. Sections were washed 3 times in TBS-T before overnight incubation in secondary antibody (Alexa488-coupled goat-anti-mouse, Molecular Probes, USA) diluted 1:500 2.5% horse serum/TBS-T. Finally, they were washed 3 times in PBS, once in PB and mounted in Vectashield (Vector Labs, CA, USA). Slices from patch-clamp experiments were processed similarly, but leaving out the incubation with primary antibody and replacing the secondary antibody with Streptavidin-Alexa488 (diluted 1:500, Molecular Probes, USA).

**Novelty-induced locomotor activity**

To assess novelty-induced hyperactivity mice were placed into novel clear plastic cages (l 43 cm, w 22 cm, h 20 cm) containing clean sawdust and locomotor activity was measured by infrared beam breaks (San Diego Instruments, San Diego, CA, US) for 2 hours in 5-minute time bins.

**Pre-pulse inhibition (PPI)**

Mice were inserted into transparent plastic tubes inside startle-response test cabinets (San Diego Instruments). After an adaptation period of 5 min at a background white noise of 65 dB, mice were subjected to a sequence of white-noise tones which started and ended by a series of five 120 dB startle-pulses, and contained 10 presentations each of startle-pulses (120 dB), pre-pulses at four levels (69 dB, 73 dB, 77 dB, 81 dB), and combinations of each pre-pulse with the startle-pulse (PPI-trials) in between. Presentations were randomized for type and occurred at random inter-trial-intervals of 10-20 s (average: 15 s). Pre-pulses lasted 20 ms, startle-pulses 40 ms, and they were separated by 100 ms intra-trial intervals in PPI-trials. The pre-pulse inhibition (PPI) was calculated for each PPI-trial individually using the average startle-response from those ten 120 dB presentations interspersed with other trials (startle-response) as the baseline and the response from the respective PPI-trial (PPI-response) according to:

PPI = 100 – (100 * PPI-response/startle-response)

PPI-values were then averaged for each pre-pulse level.

The habituation to the startle pulse was calculated from the averages of the first five (startle(start)) and the last five (startle(end)) startle-responses evoked by the respective 120 dB presentations flanking the remaining sequence.

**Elevated plus maze**

An elevated plus maze (EPM) with two opposite grey closed arms (w 7 cm, l 38 cm, h 21 cm), two open white arms (w 7cm, l 38 cm), elevated 72 cm above the ground, was used as a test of unconditioned anxiety. After a habituation time of 5 min in a novel cage in the test room, mice were placed in the center of the maze facing an open arm, and left to explore freely for 5 min. Their position was tracked with EthoVisionXT (Noldus, Wageningen, NL) and the preference for the open arms was calculated as the ratio of the total time in open arms and the time spent in open and closed arms combined (not including the center). Transition between zones was determined by the position of the center of the animal’s body.

**Hyponeophagia**

Mice were food restricted overnight prior to testing. The test apparatus consisted of a black, elevated Y-maze arm blocked off to stop access to the rest of the maze (w 9cm, l 25cm, elevated 80cm, 1 cm wall height) with a novel 20 mg sucrose pellet (TestDiet, St. Louis, MO, US) in a food well at the end of the arm. Mice were placed onto the apparatus facing away from the end of the arm and the latency to start eating was measured. Each trial lasted a maximum of 120 s, and mice were given up to 3 trials if they failed to eat initially. Latencies for mice that failed to eat in the first trial(s) were calculated by adding the total duration of trials in which they failed to eat to the latency in the trial in which they succeeded to eat; mice that never ate were scored with a latency of 360 s.

**Light/Dark-Box**

The light/dark box apparatus (Med Associates Inc., St. Albans, VT , US) consisted of an open field (w 30 cm, h 30 cm) with infrared sensors containing a black box insert covering one half of the area. The open side was brightly illuminated from above whilst the side of the black box, accessible via a 5 x 5 cm hole, was completely dark. Mice were placed into the top corner of the dark side of the apparatus and their activity was monitored for 10 minutes via infrared beam breaks using the Activity Monitor Software.

**Sucrose preference**

Mice were individually housed for this test, and the test was conducted during the dark phase. Cages contained two water bottles, placed 20 cm apart on the same side of the cage. The test was conducted over three consecutive nights. During the first night, both bottles contained normal drinking water. The contents of one bottle were replaced with 10 % sucrose (w/w; Sigma-Aldrich, Germany) in drinking water for the subsequent nights. During day time, the sucrose-containing bottle was removed and only the water bottle was left in the cage. The location of the sucrose-containing bottle was counterbalanced within each genotype, but not altered from one testing day to the next. Consumption between 7 pm and 10  am was measured for each bottle on each day and the preference for sucrose calculated as the ratio of consumption of sucrose-solution to total liquid consumption.

**Social short-term memory**

Testing was conducted in a 3-chamber apparatus as described previously ^11^, at low light levels (ca. 1 lux). CD1-mice of the same sex and weight (± 10%) were used as stimulus mice and were habituated to the apparatus and the small enclosures over 3 days before testing commenced. The test consisted of four phases, between which animals were enclosed in the central chamber but not removed from the apparatus: (i) habituation to the central chamber of the apparatus (5 min), (ii) habituation to all three empty chambers (10 min), (iii) social exposure (10 min) and (iv) social memory (10 min). During the social exposure phase a test mouse was presented with two metal cage enclosures in the far corners of the peripheral chambers, one of which contained a CD1 stimulus mouse. For the social memory phase, the empty enclosure was replaced by a third enclosure containing a CD1 stimulus mouse from a different home cage. The locations of the stimulus mice were counterbalanced with respect to both, their location in phase (iii) and the conduct of a side-swap in phase (iv) within each genotype. Interactions were scored as entries into and time spent in a 2 cm circumference of the metal cages by automated tracking in AnyMaze (San Diego Instruments). Entries into this interaction zone were registered if 30 % of the animal’s body is present within the zone, and exits were scored if less than 20 % was present in the zone. Animals were excluded from the analysis if they displayed an interaction time of < 180 s in the social exposure phase preceding the social memory test phase in order to ensure familiarity with the first stimulus mouse.

**Novel object recognition (object short-term memory)**

Mice were habituated to a dark grey open field (40 x 40 cm, 25 cm wall height) 3 times for 5 min per day for 2 days prior to testing. On the test day mice were exposed to two identical copies of object X for 10 minutes. Mice were then removed from the open field for 2 min, during which the initial objects were replaced by an identical copy of object X and a novel object Y. Mice were then reinserted into the open field and left to explore for 5 min ^12^. Direct exploration of the objects was manually scored by the experimenter blinded for genotype, and videos were recorded using AnyMaze (San Diego Instruments). Objects and the arena were cleaned with 70 % ethanol and water between animals. The identity of the first object and its position in the arena were counterbalanced within each genotype. The preference for the novel object was calculated as the ratio of the time spent with the novel object divided by the time spent with both objects combined.

**Spatial novelty preference (Y-maze, spatial short-term memory)**

Spatial novelty preference was assessed using a clear Perspex Y-maze (each arm: w 8 cm, l 30 cm, h 20 cm) as previously described ^13^. To promote exploratory behaviour the maze was scattered with a mixture of clean and dirty sawdust (3:1) from the cages of unfamiliar mice of the same sex. In the sample trial mice were placed in the start arm and allowed to explore the maze for 5 minutes with either the left or right goal arm blocked off (counterbalanced within genotype). Mice were then removed from the maze for an intra-trial interval (ITI) of 1 minute during which the sawdust was mixed and re-distributed around the maze and the divider removed. In the choice phase mice were given 2 minutes to explore the entire Y maze. The location of the mouse was tracked with EthoVisionXT (Noldus). The preference for the novel goal arm was calculated as the ratio of the time spent in the novel arm divided by the time spent in both choice arms combined.

**Rewarded alternation on the T-maze**

Rewarded alternation was tested in a wooden elevated T-maze painted grey (start arm: w 10 cm, l 47 cm; goal arms: w 10cm, l 35cm; wall-height 10 cm; elevated 100 cm above ground) with metal food wells fixed to the end of each goal arm. Prior to testing animals were put on a food-restricted diet to limit them to 85-90 % of their free-feeding weight. Mice were then habituated to the maze and the condensed milk reward (mixed 50:50 with drinking water), initially in groups and then individually. During training and testing, each trial consisted of a sample and a choice run. In the sample run mice were placed in the start arm facing the experimenter and forced into one of the goal arms to gain a reward. The forced direction was randomly assigned, with no more than three consecutive trials in the same direction, and overall equal numbers of left and right allocations. Mice were then removed from the maze for a defined intra-trial interval (1 s, 5 s or 15 s, as indicated), during which the door from the second goal arm was removed. For the choice run mice were returned to the maze start arm facing the investigator and allowed to make a free choice between both goal arms, of which the previously blocked (unvisited) one was rewarded. A decision was counted when the mouse had crossed the T-junction and all four paws were within a goal arm. Mice received 3 days of training with an inter-trial interval of 6-8 minutes (round robin regime) and an intra-trial interval of 5 seconds. After training mice were tested with different intra- and inter-trial intervals (as indicated in the figure) to manipulate the difficulty of the task and to replicate conditions used in related previous studies ^4,14^.

**Spatial reference and reversal learning**

Food deprivation, maze adaptation and reward was as described for the rewarded alternation task above. An elevated grey wooden plus maze with 10 cm wall height and 10 cm inner arm width was used; arm-length was 35 and 40 cm for goal and start arms, respectively. For each mouse, counterbalanced for genotype, one goal arm location (as defined by allocentric extra-maze cues in the room) was assigned as rewarded. The maze was rotated by 180° once per day to prevent association of subtle inner-maze cues with the reward. On every trial the start arm was pseudo-randomly chosen from the two start arms of the maze in order to prevent the subjects from using an egocentric strategy to solve the task. Mice were trained to a criterion of correct choices in 17/20 (85 %) consecutive trials. Subsequently mice were subjected to a block of 10 trials, during which reward was delivered after the choice was made, to ensure they were not solving the task simply by smelling the reward. Afterwards, the location-reward association was reversed and training continued until criterion to assess reversal learning.

**5-choice-serial-reaction-time task (5-CSRTT)**

The 5-CSRTT was conducted similar to previous descriptions ^15,16^. Mice were handled and baseline weights measured for 3 days. They were then accustomed to the milk reward used later for the training (Yazoo® Strawberry Milkshake) in their homecages and food-deprived to attain ca. 90% of baseline weight. Males were subsequently brought down to ca. 85 % baseline weight bc. of low response rates at higher weights, while females gradually assumed 95-100 % of original weight during the ca. 2 months of training accounting for normal growth curves. Females still responded significantly more and performed better throughout (see Table S1). Weights were well controlled throughout and did not differ between genotypes. Training was performed in 4 operant chambers with 5-CSRTT walls (ENV307-W, ENV115-C; Med Associates), illuminated by a light unless otherwise specified and situated in standard ventilated sound-attenuating cubicles. Mice were trained to respond to light stimuli for 6-10 sessions depending on performance levels: trials started with the presentation of a milk reward in a receptacle opposite the 5-CSRTT wall through operation of a dipper by a solenoid. Upon collection of the reward, all 5 lights in the wall were switched on until the mouse poked into one of the illuminated holes which triggered the presentation of another reward whose collection then started the next trial.

Once all mice achieved more than 40 nosepoke/reward-collection cycles the 5-CSRTT was started. As before, each session started with the presentation of a milk reward whose collection triggered an inter-trial interval period (ITI) after which one of the 5 lights at the 5-CSRTT wall was switched on for a limited period of time (stimulus duration, SD). Mice were given a limited period of time (limited hold, LH), which started with the onset of the light stimulus to respond with a nosepoke into the hole that was indicated by the light. Successful nosepokes terminated the light presentation (if still active) and triggered the delivery of a new milk reward whose collection started the next trial after 3 s. Nosepokes into the indicated hole were counted as correct (#C), nosepokes into any of the other holes as incorrect responses (#I). Nosepokes prior to stimulus onset (i.e. within the ITI period) were counted as premature responses (#Pre), repeated nosepokes into the correct hole as perseverative responses (#Prs), and failure to respond within the limited-hold time as omissions (#O). Incorrect and premature responses as well as omissions were punished by a period of darkness (house-light off for 5 s, time-out) immediately followed by a new ITI (i.e. without delivery of a reward). Relative measures were calculated as follows:

% accuracy = 100 * #C/(#C + #I)

% correct = 100 * #C/(#C + #I + #O)

% omissions = 100 * #O/(#C + #I + #O)

% premature = 100 * #Pre/(#C + #I + #Pre + responses during time-outs) = 100 * #Pre/(total number of nosepokes)

% perseverative = 100 * #Prs/(total number of nosepokes)

Mice were trained and tested in 6 stages. The parameters defining the stages, as well as the transition to the next stage, were as follows (s, seconds):

|  | **Parameters of training** | | | **Criteria to transition (on 2 consecutive days)** | | | |
| --- | --- | --- | --- | --- | --- | --- | --- |
| **Stage** | **SD (s)** | **LH (s)** | **ITI (s)** | **# correct** | **% correct** | **% accuracy** | **% omissions** |
| 1 | 20 | 30 | 2 | >= 30 | >= 30 | - | - |
| 2 | 10 | 30 | 2 | >= 50 | >= 50 | - | - |
| 3 | 8 | 20 | 5 | >= 50 | >= 50 | - | - |
| 4 | 4 | 10 | 5 | >= 50 | - | >= 80 | <= 50 |
| 5 | 4 | 7 | 5 | >= 50 | - | >= 80 | <= 40 |
| 6 | 1 | 7 | 5 | n.a. | n.a. | n.a. | n.a. |

Data from the first two days in each stage were averaged for each mouse, and performance in each of the relevant parameters analysed with repeated-measures ANOVA performed on those averages of the six stages. Measures of multiple behavioural functions were determined as follows: attention, % accuracy, % correct; perseveration, % perseverative; impulsivity, % premature; motivation and locomotion, reward latency (i.e. time needed to collect a reward after a correct nosepoke).

**MK-801 studies**

MK-801 ((+)-MK-801 maleate, Tocris, Bristol, UK) was injected i.p. as a solution of 0.025 mg/ml (for doses 0.1 and 0.15 mg/kg) or 0.05 mg/ml (for doses 0.2, 0.4 and 0.5 mg/kg) in sterile saline vehicle. Control-mice received vehicle injections of corresponding volumes. Experiments involving MK-801-injections were distributed across cohorts, so that mice had received either none or only one (low-dose) injection of MK-801 before each experiment, apart from the T-maze.

For the **T-maze** mice were trained as well as tested with massed trials with 5 s intra- and 45 s inter-trial-intervals. All mice were trained for at least 3 days and to a criterion of 7/10 correct choices on two consecutive days before receiving injections of MK-801 or vehicle. Doses were applied in a rising order and a drug-session at each dose was accompanied by a vehicle session conducted before or after in a counterbalanced fashion (within-subject design). Blocks of ten massed trials were run starting 30 min post injection. For **sucrose preference** under MK-801 mice were tested at 0.1 mg/kg or 0.15 mg/kg in a within-subjects design after receiving two nights of habituation with one water- and one 1%-sucrose-bottle. MK-801/vehicle injections and/or testing started at the onset of the dark-phase and measurements of consumption were taken for the immediately following periodof 2.5 h. Sucrose bottles remained in the cage for the whole night, but were removed 2-3 h after the onset of the light phase leaving only the water bottle for the rest of the light phase.

For **locomotor-activity (LMA)-experiments** involving MK-801, animals were scored by experienced raters blind for genotype online to provide a view from the side as well as from top. The time spent in states of catalepsy, unbalanced walking or standing, and stereotypies (mostly circling) was scored as described in the Results section. Episodes of normal sitting, resting, walking or exploring were not scored separately.

For **rotarod** experiments assessing motor coordination under MK-801, mice were placed on a wooden rod and transferred to the rotarod (Ugo Basile, Gemonio, IT) rotating at 4 rpm. After an accommodation period of 10 s, the angular speed was accelerated linearly to 20 rpm. The time of falling was recorded. If mice fell during the accommodation period (10 s), the trial was recorded as a failure and two more chances were given immediately afterwards to complete the task. Mice were tested 30 and 60 min after the injection of vehicle or MK-801 within the same trial. Females were tested at a lower dose (0.15 mg/kg) because of the failure of some female knockouts to be transferred to the rotarod at all when injected with 0.2 mg/kg.

Rotarod and LMA-experiments were conducted in mixed-sex cohorts and in a between-subjects design.

**Electrophysiology *in vivo***

Mice were anaesthetized and inserted into a stereotactic frame. Straightened polyimide-insulated tungsten wires of 50 µm inner diameter (Wiretronic, CA, USA) were inserted into medial prefrontal cortex (+1.7 mm AP, +0.35 mm ML, 1.9 mm below pia, targeting mainly IL), dorsal hippocampus (-2 mm AP,  +1.5 mm ML, 1.4 mm below pia) and ventral hippocampus (-3.2 mm AP, 2.9 mm ML, 3.4 mm below pia) of the right hemisphere, largely following previous studies ^17,18^. As in those studies, a reference electrode (stainless steel wire, 125 µm diameter, Advent Research Materials, UK) was inserted over the posterior part of frontal cortex and a ground screw was lowered into the cerebellum, both contra-laterally with respect to recording electrodes.

Starting after a week of recovery, recordings were conducted in awake mice moving freely in novel cages. Data was acquired at 15 kHz sampling rate and a 0.1-300 Hz bandpass filter using an RHD2132 amplifier board (Intan Technologies, CA, USA), an Open E-Phys USB interface board and the Open EPhys acquisition software (Open E-Phys, MA, USA). Data was exported into MatLab (Mathworks, MA, USA), decimated down to 1 kHz, and further analysed with custom-written procedures in IgorPro (Wavemetrics, OR, USA). The IgorPro procedure “PowerSpectralDensity.ipf” was used to calculate power-spectra using Hann-windowing and a segment length of 8192 points. Power-spectra were integrated within the following frequency bands to analyse genotype- and drug-related differences: delta (1-5 Hz) including higher delta (3-5 Hz), theta (5-10 Hz), gamma (30-80 Hz) including lower gamma (30-50  Hz).

**Electrophysiology *in vitro (patch-clamp)***

Animals were transcardially perfused with a room-temperature solution containing (in mM): N- Methyl-D-glucamine, 92; KCl, 2.5; NaH_2_PO_4_, 1.25; Thiourea, 2; Ascorbic acid, 5; Na-Pyruvate, 3; MgCl_2_, 10; D-Glucose, 25; NaHCO_3_, 30; CaCl_2_, 0.5; Sucrose, 1, and horizontal neocortical slices were prepared. The extracellular perfusion solution contained (in mM): NaCl, 119; KCl, 2.5; CaCl_2_, 0.5; MgSO_4_, 1.3; MgCl, 2; NaH_2_PO_4_, 1.25; NaHCO_3_, 25; Glucose, 10. 100 μM Picrotoxin was added to block GABA_A_-receptors. PV-cells were identified according to red (tdTomato) fluorescence and recorded in voltage-clamp mode and whole-cell patch-clamp configuration. Pipette solution contained (in mM): CsGlu, 125; HEPES, 10; Na-Phosphocreatine, 10; NaCl, 8; Na-GTP, 0.33; EGTA, 0.2; TEA-Cl, 5; Mg-ATP, 4. A field extracellular stimulation (20-320 μA, 100 μs) was conducted at a distance of 200 – 500 μm from the recorded cells, every 40 s. Stimulation-evoked AMPA-receptor-mediated currents were recorded as inward currents at a holding potential of -70 mV. Subsequently, 50 μM NBQX was added to the extracellular solution to block AMPA/Kainate-type glutamate receptors, and NMDA-receptor-mediated currents were recorded as outward currents at a constant holding potential of +40 mV. Neurons were held at +40 mV for at least 1 min before measuring the NMDA component to minimise contaminating outward current and ensure unblocking of the NMDA-receptor channel.

**Analysis**

Time-series and within-subject data was analysed using repeated measures ANOVA. Other data with multiple independent variables were assessed with univariate ANOVA followed by T-Tests. Data-sets with a single independent variable were analysed by T-Tests or Mann-Witney-U-Test (abbr. M.W.U-Test) as appropriate. Two-sided tests were used throughout. Significance level was p < 0.05 throughout. Except for locomotor interval graphs, data are plotted with error bars as 95 % confidence intervals placed symmetrically around the mean for ease of pairwise and 1-sample comparisons. Error-bars in locomotor interval plots represent S.E.M. for clarity of presentation.

## Supplementary videos

**Supplementary video 1**

A control mouse displaying unbalanced walking, running, rearing and standing (tumbling) induced by MK-801.

**Supplementary video 2**

A *Grin1^ΔPV^* knockout displaying catalepsy induced by MK-801.

**Supplementary video 3**

A *Grin1^ΔPV^* knockout displaying direct switches between catalepsy and stereotypic circling induced by MK-801.

**Supplementary video 4**

A *Grin1^ΔPV^* knockout displaying direct switches between catalepsy and stereotypic head-shakes induced by MK-801.

## Supplementary References

1 Hippenmeyer S, Vrieseling E, Sigrist M, Portmann T, Laengle C, Ladle DR *et al.* A Developmental Switch in the Response of DRG Neurons to ETS Transcription Factor Signaling. *PLoS Biol* 2005; **3**: e159.

2 Belforte JE, Zsiros V, Sklar ER, Jiang Z, Yu G, Li Y *et al.* Postnatal NMDA receptor ablation in corticolimbic interneurons confers schizophrenia-like phenotypes. *Nat Neurosci* 2010; **13**: 76–83.

3 Fuchs EC, Zivkovic AR, Cunningham MO, Middleton S, Lebeau FE, Bannerman DM *et al.* Recruitment of parvalbumin-positive interneurons determines hippocampal function and associated behavior. *Neuron* 2007; **53**: 591–604.

4 Korotkova T, Fuchs EC, Ponomarenko A, von Engelhardt J, Monyer H. NMDA receptor ablation on parvalbumin-positive interneurons impairs hippocampal synchrony, spatial representations, and working memory. *Neuron* 2010; **68**: 557–69.

5 Carlen M, Meletis K, Siegle JH, Cardin JA, Futai K, Vierling-Claassen D *et al.* A critical role for NMDA receptors in parvalbumin interneurons for gamma rhythm induction and behavior. *Mol Psychiatry* 2012; **17**: 537–548.

6 Saunders JA, Tatard-Leitman VM, Suh J, Billingslea EN, Roberts TP, Siegel SJ. Knockout of NMDA receptors in parvalbumin interneurons recreates autism-like phenotypes. *Autism Res Off J Int Soc Autism Res* 2013; **6**: 69–77.

7 Dang MT, Yokoi F, Yin HH, Lovinger DM, Wang Y, Li Y. Disrupted motor learning and long-term synaptic plasticity in mice lacking NMDAR1 in the striatum. *Proc Natl Acad Sci* 2006; **103**: 15254–15259.

8 Tsien JZ, Huerta PT, Tonegawa S. The essential role of hippocampal CA1 NMDA receptor-dependent synaptic plasticity in spatial memory. *Cell* 1996; **87**: 1327–38.

9 Niewoehner B, Single FN, Hvalby ?, Jensen V, Meyer zum Alten Borgloh S, Seeburg PH *et al.* Impaired spatial working memory but spared spatial reference memory following functional loss of NMDA receptors in the dentate gyrus. *Eur J Neurosci* 2007; **25**: 837–846.

10 Crawley JN. Behavioral phenotyping of transgenic and knockout mice: experimental design and evaluation of general health, sensory functions, motor abilities, and specific behavioral tests. *Brain Res* 1999; **835**: 18–26.

11 Moy SS, Nadler JJ, Perez A, Barbaro RP, Johns JM, Magnuson TR *et al.* Sociability and preference for social novelty in five inbred strains: an approach to assess autistic-like behavior in mice. *Genes Brain Behav* 2004; **3**: 287–302.

12 Sanderson DJ, Hindley E, Smeaton E, Denny N, Taylor A, Barkus C *et al.* Deletion of the GluA1 AMPA receptor subunit impairs recency-dependent object recognition memory. *Learn Mem* 2011; **18**: 181–190.

13 Sanderson DJ, Good MA, Skelton K, Sprengel R, Seeburg PH, Rawlins JNP *et al.* Enhanced long-term and impaired short-term spatial memory in GluA1 AMPA receptor subunit knockout mice: Evidence for a dual-process memory model. *Learn Mem* 2009; **16**: 379–386.

14 Carlén M, Meletis K, Siegle JH, Cardin J a, Futai K, Vierling-Claassen D *et al.* A critical role for NMDA receptors in parvalbumin interneurons for gamma rhythm induction and behavior. *Mol Psychiatry* 2012; **17**: 537–48.

15 Bari A, Dalley JW, Robbins TW. The application of the 5-choice serial reaction time task for the assessment of visual attentional processes and impulse control in rats. *Nat Protoc* 2008; **3**: 759–767.

16 Papaleo F, Erickson L, Liu G, Chen J, Weinberger DR. Effects of sex and COMT genotype on environmentally modulated cognitive control in mice. *Proc Natl Acad Sci* 2012; **109**: 20160–20165.

17 O’Neill P-K, Gordon JA, Sigurdsson T. Theta Oscillations in the Medial Prefrontal Cortex Are Modulated by Spatial Working Memory and Synchronize with the Hippocampus through Its Ventral Subregion. *J Neurosci* 2013; **33**: 14211–14224.

18 Sigurdsson T, Stark KL, Karayiorgou M, Gogos JA, Gordon JA. Impaired hippocampal-prefrontal synchrony in a genetic mouse model of schizophrenia. *Nature* 2010; **464**: 763–7.
